# Quantitative Prediction of Steatosis in Patients with Non-Alcoholic Fatty Liver by Means of Hepatic MicroRNAs Present in Serum and Correlating with Hepatic Fat

**DOI:** 10.3390/ijms23169298

**Published:** 2022-08-18

**Authors:** Guillermo Quintás, Florian Caiment, Iván Rienda, Judith Pérez-Rojas, Eugenia Pareja, José V. Castell, Ramiro Jover

**Affiliations:** 1 Unidad Analítica, Health Research Institute Hospital La Fe, 46026 Valencia, Spain; 2 Health and Biomedicine, LEITAT Technological Centre, 08005 Barcelona, Spain; 3 Department of Toxicogenomics, School of Oncology and Developmental Biology (GROW), Maastricht University, 6229-ER Maastricht, The Netherlands; 4 Pathology Department, Hospital Universitario y Politécnico La Fe, 46026 Valencia, Spain; 5 Servicio de Cirugía General y Aparato Digestivo, Hospital Universitario Dr. Peset, 46017 Valencia, Spain; 6 Unidad Mixta de Investigación en Hepatología Experimental, Health Research Institute Hospital La Fe, 46026 Valencia, Spain; 7 Departamento de Bioquímica y Biología Molecular, Facultad de Medicina, Universidad de Valencia, 46010 Valencia, Spain; 8 Centro de Investigación Biomédica en Red de Enfermedades Hepáticas y Digestivas (CIBERehd), Instituto de Salud Carlos III, 28029 Madrid, Spain

**Keywords:** non-alcoholic fatty liver disease, steatosis quantification, circulating miRNAs, non-invasive, NAFLD patient screening, NAFLD patient stratification

## Abstract

Non-alcoholic fatty liver disease (NAFLD) is the most prevalent form of chronic liver disease worldwide, but a reliable non-invasive method to quantify liver steatosis in primary healthcare is not available. Circulating microRNAs have been proposed as biomarkers of severe/advanced NAFLD (steatohepatitis and fibrosis). However, the use of circulating miRNAs to quantitatively assess the % of liver fat in suspected NAFLD patients has not been investigated. We performed global miRNA sequencing in two sets of samples: human livers from organ donors (*n* = 20), and human sera from biopsy-proven NAFLD patients (*n* = 23), both with a wide range of steatosis quantified in their liver biopsies. Partial least squares (PLS) regression combined with recursive feature elimination (RFE) was used to select miRNAs associated with steatosis. Moreover, regression models with only 2 or 3 miRNAs, with high biological relevance, were built. Comprehensive microRNA sequencing of liver and serum samples resulted in two sets of abundantly expressed miRNAs (418 in liver and 351 in serum). Pearson correlation analyses indicated that 18% of miRNAs in liver and 14.5% in serum were significantly associated with the amount of liver fat. PLS-RFE models demonstrated that 50 was the number of miRNAs providing the lowest error in both liver and serum models predicting steatosis. Comparison of the two miRNA subsets showed 19 coincident miRNAs that were ranked according to biological significance (guide/passenger strand, relative abundance in liver and serum, number of predicted lipid metabolism target genes, correlation significance, etc.). Among them, miR-10a-5p, miR-98-5p, miR-19a-3p, miR-30e-5p, miR-32-5p and miR-145-5p showed the highest biological relevance. PLS regression models with serum levels of 2–3 of these miRNAs predicted the % of liver fat with errors <5%.

## 1. Introduction

Non-alcoholic fatty liver disease (NAFLD), with a global prevalence of ~25% [1,2], is one of the most important causes of liver disease [3] and will likely emerge as the leading cause of end-stage liver disease and transplantation soon. Its growing incidence in western countries is putting increasing pressure on healthcare systems.

The pathogenesis of NAFLD is considered multifactorial, and multiple mechanisms may be triggered during the course of the disease in each patient [4,5,6]. NAFLD involves, first, the accumulation of triglycerides (TG) in the liver, which may trigger a liver inflammatory response and damage. The histologic features range from isolated hepatic steatosis foci (non-alcoholic fatty liver; NAFL) to lobular inflammation and hepatocyte ballooning, which are hallmarks of non-alcoholic steatohepatitis (NASH). NASH very frequently precedes progressive fibrosis as a hallway to cirrhosis, liver-related and cardiovascular diseases, and hepatocellular carcinoma [4,5,6].

The current gold standard in NAFLD diagnosis and prognosis is the liver biopsy, where steatosis, lobular inflammation, ballooning, and fibrosis are semi-quantitatively scored. However, this is an invasive and not risk-free procedure, with frequent sampling errors (i.e., NAFLD does not affect the liver uniformly) and complications (pain, serious bleeding, injury to other organs, and, in rare cases, fatal outcomes). Moreover, histology interpretation is strongly influenced by pathologist training and perception [7]. However, while a number of non- or minimally invasive techniques (i.e., medical imaging) can rule out advanced fibrosis or cirrhosis, no completely reliable procedure to identify and score steatosis in early NAFLD patients is available [8].

Ultrasound has been used as a screening tool for fatty liver since it is non-invasive, inexpensive, and widely accessible in the clinical setting [9,10]. Nevertheless, the sensitivity and specificity of ultrasound imaging in accurately detecting the steatosis grade is controversial. Previous studies have shown wide ranges of sensitivity of 60–94% and specificity of 66–95% in detecting fatty liver. Other studies demonstrated that ultrasound is unreliable in detecting low-grade alterations in hepatic fat content. Moreover, ultrasound cannot reliably distinguish between fibrosis and steatosis. Finally, ultrasound does not provide reproducible quantitative information, and it can be influenced by the subjectivity of the examiner [9,10].

Other more reliable, non-invasive, imaging methods have other disadvantages that limit their use as a widely applicable screening tool for this prevalent disease. Computed tomography offers a semi-quantitative method for hepatic steatosis assessment, but it is distorted by an iron overload and involves exposure to radiation, which limits its use in longitudinal studies and in children. Imaging via magnetic resonance offers high accuracy for liver fat quantification without radiation exposure, but these scanners, in contrast to ultrasonographs, are costly, resource-demanding and frequently not applicable for certain patients (young children, claustrophobia, implanted electronic devices, metal implants, etc.) [9].

Consequently, there is an unmet need for reliable, accurate, simple, cost-affordable, quantitative, and non- or minimally invasive predictive NAFLD biomarkers in the clinical setting. Some biomolecules could meet this demand. MicroRNAs (miRNAs) are small, endogenous, noncoding RNAs involved in the control of many cellular pathways through the posttranscriptional regulation of target mRNAs, which are frequently released out of the cell into circulation. Liver miRNAs are definitively involved in the pathogenesis of NAFLD/NASH at various stages of the disease [11]. Consequently, several studies aimed at identifying circulating biomarkers for NAFLD have focused on miRNAs. Surprisingly, the miRNAs found in these independent studies show a very limited overlap, and only the liver specific miR-122 was unanimously claimed as a potential circulating NAFLD/NASH biomarker. Such a lack of agreement is likely to come from the bias in miRNA pre-selection, the reduced number of miRNAs examined, different technical approaches to quantify them, small study-size populations, and no biopsy-proven NAFLD diagnosis [12]. More recently, studies using large cohorts of patients and comprehensive miRNA profiling have overcome some of the previous shortcomings [13]. Nevertheless, relevant remaining questions are whether the serum miRNAs claimed as biomarkers (solely based on their biostatistical significance) do have a significant expression in the liver and show a quantitatively strong association with the steatosis grade, which is the early hallmark of NAFLD. It also needs to be determined whether the liver contributes significantly to the serum concentration of these postulated miRNAs and whether they have a potential implication in lipid metabolism and in the pathogenesis of NAFLD. Thus, it is conceivable that for an miRNA to be a reliable biomarker of hepatosteatosis, it should be liver-enriched, regulate lipid metabolism genes and correlate with the level of intrahepatic TGs. However, to our knowledge, the many studies investigating miRNAs as biomarkers of NAFLD have generally not addressed these logical premises.

Hepatosteatosis (lipid droplets in more than 5% of hepatocytes) is considered the primary event in the onset of NAFLD and is preceded by deregulated expression of key genes and proteins. Therefore, hepatosteatosis could also be associated with changes in miRNAs that regulate the expression of genes involved in lipid metabolism that, if released properly into the blood, could become early quantitative circulating NAFLD biomarkers.

Hence, our objective was, first, to comprehensively analyze the miRNAs expressed in steatotic human livers and examine their correlation with the concentration of liver TGs; and second, to analyze all human serum miRNAs present in NAFLD patients histologically diagnosed with different steatosis grades and examine their correlation with the % of liver fat in the stained liver biopsies—all of this to identify common liver and serum deregulated miRNAs in steatotic patients, score their relevance, and develop a serum-based model with the most relevant miRNAs able to predict the % of liver fat in patients with early NAFLD.

## 2. Results

### 2.1. Human Liver and Serum MicroRNA Sequencing and Building of Steatosis Predictive Models

**Figure 1:**
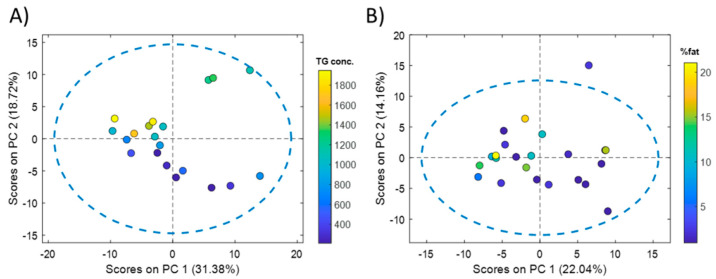
**PCA of liver and serum miRNA profiles.** PCA scores calculated from the analysis of human liver tissue (**A**) and serum (**B**) miRNA profiles. Color scales in the score plots indicate the reference liver TG concentration (µg TG/mg protein) (**A**) or the % of fat quantified in hematoxylin-eosin stained liver biopsies (**B**).

Comprehensive microRNA sequencing of 20 liver samples from cadaveric organ donors resulted in a list of 418 miRNAs detected in at least 18 samples (90%). From these 418 miRNAs, 89 (18%) showed a significant Pearson correlation with the concentration of liver TGs. Principal component analysis (PCA) showed an association between the scores of the first principal component (PC), explaining 31.4% of the variance, and the TG content (Figure 1A).

**Figure 2:**
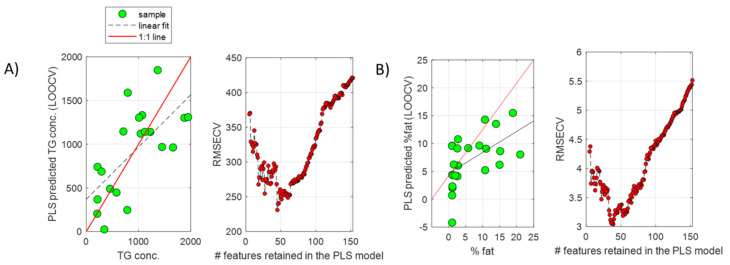
**PLS regression of liver and serum miRNA profiles.** PLS predicted TG concentrations (µg TG/mg protein) ((**A**), left) or % fat ((**B**), left) by LOOCV, and evolution of the RMSECV as a function of the number of retained features included in the PLS models during the recursive feature elimination analysis for model optimization in liver tissue ((**A**), right) or serum ((**B**), right) samples.

A PLS model was built, providing a statistically significant RMSECV = 421 (permutation test *p*-value = 0.01). The recursive feature elimination (RFE) algorithm was employed to find the subset miRNA providing the lowest RMSECV in predicting the %TG. From results depicted in Figure 2A, a subset of 50 miRNAs was selected, providing an RMSECV = 240 (57% of the error obtained using the whole miRNA set).

The same strategy was followed for the analysis of serum miRNA profiles. Whole miRNome sequencing of 23 serum samples from NAFLD patients resulted in a list of 351 miRNAs that were detected in at least 19 serum samples (82.5%). From these 351 miRNAs, 51 (14.5%) showed a significant Pearson correlation with the % of fat in the paired liver biopsies of these patients. PCA showed an association between the scores of the first PC, explaining 22.0% of the variance, and the % of fat (Figure 1B). A PLS model was built using the complete set of retained miRNAs, providing a statistically significant RMSECV = 6 (permutation test *p*-value = 0.03). Results by RFE enabled the identification of a subset of 50 miRNAs, providing an RMSECV = 3 (50% of the error initially obtained using the whole miRNA set) (Figure 2B). The comparison of the two subsets of miRNAs selected from the liver and serum datasets showed that 19 miRNAs (listed in Table 1) were coincident.

### 2.2. Biological Significance of the 19 miRNAs Identified Both in Liver and Serum That Show Correlation with Liver Steatosis

**Figure 3:**
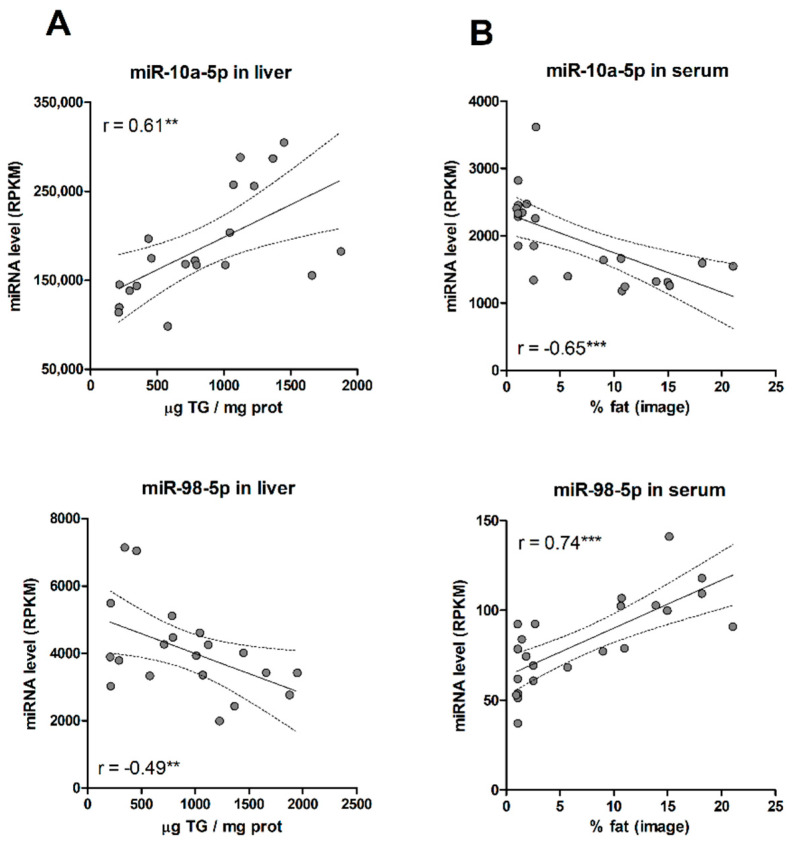
(**A**) Correlation between liver miRNA levels and tissue TG concentrations. (**B**) Correlation between serum miRNA levels and % fat in paired liver biopsies. **, *p* < 0.01; ***, *p* < 0.001.

First, most of the selected miRNAs matched the guide strand of their miRNA duplex (Table 1). The guide strand is retained in Ago proteins and stably forms the RISC complex. The other strand, known as the passenger strand (or miRNA *), is discarded and usually degraded. Nevertheless, in some cases, both arms of the duplex give rise to functional mature miRNAs that are loaded into Ago proteins. As our goal was to identify circulating miRNA biomarkers, the more abundant guided strands were preferred. Secondly, we assessed the trend and strength of the association of each miRNA with liver TGs and % of fat. We unexpectedly found that for most of the selected miRNAs (80%), the positive or negative association with steatosis in the liver usually translated into an association with the opposite trend in serum (Table 1, Figure 3). Thus, most miRNA that increased with TG levels in the liver, steadily decreased in serum with the % of liver fat, and vice versa. Results suggest that, for most miRNAs, hepatosteatosis influences the dynamics of miRNA release rather than miRNA biogenesis.

It was also important to consider the absolute value and significance of the Pearson correlation coefficient. Some serum miRNAs selected by the PLS-RFE model (i.e., 148a-3p, 660-5p, 335-5p and 17-3p) did not show a significant correlation (Table 1), and this could limit their convenience as individual predictive circulating biomarkers. To check the biological relevance of the selected miRNAs, we used the DIANA-microT-CDS [14] and the mirDIP algorithms [15] against the collection of lipid metabolism genes, as specified by REACTOME Id: R-HSA-556833, containing 749 proteins from 730 genes. Many of the selected miRNAs were predicted to regulate numerous lipid metabolism genes, but some of them (e.g., 191-5p, 769-5p, 660-5p, 30a-3p, and 136-3p) had a very limited impact on these genes (Table 1). Finally, we assessed the abundance of these miRNAs in the liver. It is conceivable that a circulating miRNA biomarker of hepatosteatosis will be significantly expressed in and released from the liver. Moreover, it is likely that liver-specific miRNAs will be diluted in the blood, resulting in high liver level/serum level ratios. The more tissues contributing to the serum concentration of a given miRNA, the lower the liver/serum ratio will be. Liver-enriched miRNAs 122-5p and miR-192-5p exhibit liver/serum ratios between 300 and 3000. We observed low liver/serum ratios with several of the selected miRNAs, such as let-7d-5p, let-7b-5p, 191-5p or 140-3p (Table 1).

### 2.3. PLS Model Based on Only 2-3 Serum miRNAs to Predict % of Fat in NAFLD Patients

**Figure 4:**
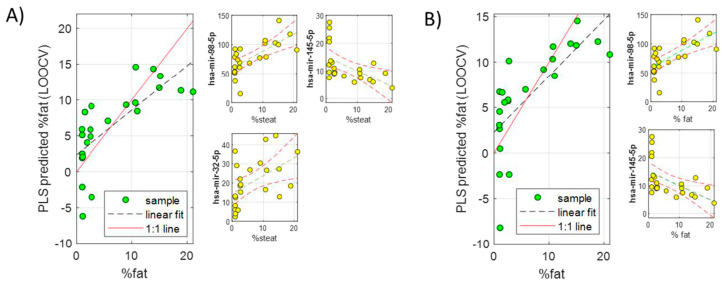
Predicted liver fat % by LOOCV in two PLS models built using two serum miRNA subsets including three (**A**) or two (**B**) miRNAs.

A series of PLS models including two or three miRNAs from the subset of 19 common miRNAs (quantified in serum samples) were investigated by exhaustive feature selection. The performance for the accurate prediction of liver fat % was again evaluated. The combinations of 2–3 miRNAs with higher biological significance and more accurate prediction of fat % (lower RMSECV) were finally selected (Table 2, Figure 4). Therefore, according to these models, it would be possible to predict the % of fat in the liver of NAFLD patients by quantifying 2–3 miRNAs in the serum with an error of around 4–5%. This could become a very useful tool to screen early NAFLD in the general population and stratify patients according to their liver fat %.

## 3. Discussion

A group of experts has recently proposed a change in nomenclature from non-alcoholic fatty liver disease (NAFLD) to metabolically associated fatty liver disease (MAFLD). The new term better reflects the pathophysiology of NAFLD as a metabolically driven disease, shifting from “exclusion” criteria to “positive” diagnosis criteria. One important additional difference is that NAFLD diagnosis is based primarily on histological criteria, being steatosis-graded by anatomical pathologists. However, for MAFLD, steatosis may be diagnosed non-invasively with either imaging techniques or biomarkers [16]. In this regard, ultrasonography, contrary to other imaging techniques, is widely available and could be the first-line diagnostic tool for assessing suspected hepatic steatosis or a screening tool for asymptomatic NAFLD [17]. However, while ultrasonography could be reliable at detecting moderate steatosis, its sensitivity is thought to be poor when <20–30% of hepatocytes are steatotic [17]. For instance, in a comparative study of different imaging techniques, ultrasonography showed the lowest sensitivity (65%) and specificity (77%) in detecting ≥5% histologically defined hepatic steatosis [18]. In addition, ultrasonography usually reports steatosis on a semiquantitative scale (normal, mild, moderate, and severe). Thus, the complementation of widely available imaging techniques along with circulating biomarkers could increase accuracy in steatosis diagnosis, and with a quantitative score.

Several classic biomarkers are included in algorithms intended to score steatosis. However, most of these biomarkers are nonspecific enzymes unrelated to liver TG accumulation. The hepatic steatosis index, NAFLD liver fat score, and fatty liver index include typical liver enzymes such as hepatic transaminases or γGT. However, it is well known that normal enzyme levels can be found in NAFLD patients representing the entire spectrum of disease severity, from simple steatosis to advanced NASH [19].

The search for novel useful biomarkers for early NAFLD that could routinely and economically be applied to patients to diagnose, screen, and/or monitor the extent and progress of NAFLD led us to examine in detail biomolecules that originated in the liver, are present in patient’s sera, and could account for the % of fat in the liver. By using two separate cohorts, we identified, from the large number of miRNAs present in liver tissue and sera of individuals with a variable grade of steatosis, those having a stronger association with the % of liver fat and mechanistically involved in lipid metabolism regulation.

A key feature making serum miRNAs excellent potential biomarkers is their stability. They are resistant to different external insults because they do not circulate as free RNA; instead, they are encapsulated in membranous vesicles (micro-vesicles, exosomes, apoptotic bodies), complexed to RNA-binding proteins (e.g., Ago2) or associated with lipoproteins, which protect them from endogenous RNases. Another aspect making miRNA molecules attractive biomarkers is that the RT-qPCR technique used for their detection is extremely sensitive and cost-effective [20].

Several previous studies have also investigated miRNA as biomarkers for NAFLD. In a previous study, we attempted to validate many of these miRNAs postulated in previous studies, but the rate of success was very low. Our results suggested that measurement design, technical and methodological differences, the pre-selection of miRNA to be measured, and the reduced number of miRNAs examined likely limited reproducibility [12]. In the present study, a comprehensive miRNAseq encompassing all of the expressed miRNAs in serum and liver tissue was performed. Another important bias is that in some studies, NAFLD diagnosis is not biopsy-proven, thus opening the possibility that false positive and negative patients could be inadvertently included. Our study is the first including not only biopsy-proven patients but also patients with an accurate quantification of steatosis either by biochemical methods (µg TG/mg liver protein) or by image quantifications (based on the % of the area occupied by lipid droplets in the hematoxylin-eosin stained liver biopsy). As an important contribution to minimize subjective biasing in the score of steatosis, we had high-quality and sufficiently large biopsies analyzed by the same pathologist in a blind manner; that is, she was not aware of the clinical features and diagnosis of the patients at the time of examination. On top of that, steatosis was estimated not only visually but also by computer analysis of images taken from various fields of hematoxylin-eosin stained tissue sections. This image quantification was based on previous studies [21] and validated before being applied in this work by comparing computer data analysis and the lipid content (biochemically determined) from the same liver samples.

Out of the large number of miRNAs identified in liver tissue and serum, a much smaller number fulfilled the criteria we set ourselves, i.e., (a) be liver-enriched, (b) be involved in the regulation of lipid metabolism genes, and (c) correlate significantly with the level of intrahepatic lipids. Only a few of the model-selected miRNAs met these criteria.

The relevance of some of the miRNAs selected after our regression modeling and biological fine-tuning was reinforced by previous studies searching for miRNA biomarkers. Thus, miR-23a-3p and miR-19a-3p were found to be deregulated (with an opposite trend) in the serum of patients with NASH [22]. Similarly, miR-19a and b were both upregulated in the serum of simple steatosis NAFLD patients [23], whereas in a different study, miR-30a-3p was also substantially upregulated in NAFLD patients, and its suppression attenuated hepatic steatosis in HepG2 cells [24]. In an animal model, miR-145-5p increased in steatotic liver in response to a high-fat diet [25]. Moreover, ingestion of a high-fat high-saturated meal was able to deregulate miR-145-5p in human serum [26].

Serum levels of several members of the group of 19 miRNAs (miR-145-5p, miR-23a-3p, miR-148a-3p, and miR-191-5p) were also significantly altered in patients with metabolic syndrome or with NAFLD-associated metabolic alterations [27].

Several of the miRNAs proposed as steatosis biomarkers have demonstrated links to key important lipid metabolism pathways. Thus, miR-98-5p inhibits PGC-1β mRNA expression in the liver, and PGC-1β plays an important role in the regulation of hepatic lipogenesis by activating genes such as Scd1 and Fas [28]. The decrease in liver miR-98-5p observed in our study would likely result in more PGC-1β and lipogenesis.

In a different study, miR-32-5p targeted KLF3, which in turn represses lipogenic regulators such as PPARγ and C/EBPα [29]. In this case, the decrease in liver miR-32-5p observed in our study will result in higher KLF3 and lower lipogenesis as a likely compensatory response.

Another recent study showed that hepatocytes release let-7b-5p after stimulation with palmitic acid by a TGFβ-dependent mechanism. Moreover, let-7b-5p overexpression increased hepatocyte fatty acid transport [30]. In our study, this miRNA was upregulated both in the liver and serum.

Nevertheless, the potential utility of the proposed novel miRNA biomarkers might be limited by other factors, such as the complex contribution of other co-morbid pathologies, particularly obesity, T2D and dyslipidemia. Further improvements to give confidence to our findings would include performing a paired analysis of liver and serum miRNAs from the same cohort of patients, as well as running a prospective study to monitor the progression of the disease by miRNA analysis, medical imaging, and whenever possible, liver biopsy. This clinical study is currently ongoing.

Moreover, due to the limited sample size of the two study cohorts, further efforts will be required to validate our results in much larger sample size cohorts. However, if these results are confirmed in future studies, patients with NAFLD may benefit from diagnostic or surveillance programs based on minimally invasive miRNA biomarkers.

## 4. Materials and Methods

### 4.1. Patients and Human Samples

Twenty human liver samples were obtained from organ donors who were primarily assigned to liver transplantation, but their livers failed to be transplanted and were donated to research (Biobank Hospital La Fe, Valencia, Spain). Histological analyses were not performed on these livers, but we did perform biochemical analyses of TG and total liver lipids and found a wide range of variation (Table 3). Anthropometric and analytical characteristics of these liver donors have been described elsewhere [31] and are summarized in Table 3.

Cadaveric organ donors comprised 14 men and 6 women, and their causes of death included cerebrovascular accident (either hemorrhagic or ischemic, *n* = 15) or cranioencephalic traumatism (*n* = 5). All of them were not harboring any infectious disease and tested negative for human immunodeficiency and hepatitis viruses. Livers were obtained in the operating room and transported in a cold preservation solution (University of Wisconsin solution). After the reception, and once rejected for transplantation, livers were immediately cut into small pieces, frozen in liquid nitrogen, and stored at −80 °C until use. In this cohort of donors, the hepatic levels of intrahepatic TG and total lipids covered a wide range (10–20-fold variation). The predominant lipids in the liver were TG and consequently, the correlation between total lipids and TG was very high (R Pearson = 0.91). We also observed significant variability in serum bilirubin and transaminases (AST and ALT) (Table 3). However, they did not show any correlation with liver TG levels, thus ruling them out as collinear confounding variables. It is important to remark that biochemical data from these donors were not necessarily obtained in standard conditions, and some parameters might be influenced by other factors, such as the timing of blood extraction and cerebral death.

Human sera were collected from 23 well-characterized NAFLD patients, covering all grades of steatosis as confirmed by percutaneous liver biopsy. The NAFLD patients did not consume alcohol regularly (less than 20 g/day), and other potential causes of liver disease (viral, autoimmune, hepatotoxic drugs, iron overload, Wilson’s disease, etc.) were excluded. Hematoxylin-eosin and Masson’s trichrome-stained paraffin-embedded liver biopsy sections were examined and interpreted by the same experienced hepatopathologist (J.P.-R.), who was unaware of the patients’ clinical data. All liver biopsies examined showed more than 10 complete portal tracts. Steatosis along with ballooning, lobular inflammation and fibrosis were assessed as outlined by Brunt et al. [32]. Disease severity was scored according to both NAS [32,33] and SAF [34,35] systems. Steatosis, as % of fat in the biopsy, was automatically quantified by image analysis at 20× resolution of hematoxylin-eosin stained sections in MATLAB 2021b (Mathworks Inc., Natick, MA, USA) using in-house written scripts and the Image Processing Toolbox (MATLAB), following a strategy similar to that previously described by Munsterman et al. [21]. Anthropometric and analytical characteristics of these NAFLD patients have been described elsewhere [12] and are summarized in Table 4. Anticoagulated venous blood was extracted between 8 and 10 am, after overnight fasting, by the time of liver biopsy. Blood was collected in siliconized tubes and centrifuged at 2500× *g* for 10 min. Serum samples were stored at −80 °C.

### 4.2. Quantification of Intrahepatic TG and Total Lipids

Homogenates from human livers were extracted with a methanol-chloroform mixture and evaporated under nitrogen as described elsewhere [36]. TG and total lipid concentrations were measured in the lipid residue using colorimetric kits from Spinreact (Gerona, Spain) based on the GPO-POD enzymatic method (#1001311) and the sulfo-phospo-vanillin reaction (#1001270), respectively. Protein concentration was determined using the Protein Assay Kit from Bio-Rad Laboratories (Madrid, Spain).

### 4.3. RNA Isolation, Small RNA Library Preparation and miRNAseq Analysis

Total RNA was isolated from liver and serum samples using the miRNeasy mini kit (Qiagen Westburg BV, Leusden, The Netherlands) according to the manufacturer’s protocol, followed by a DNAse I treatment (Qiagen Inc., Venlo, The Netherlands‎). RNA concentration and quality were measured using a BioAnalyzer system (Agilent Technologies, Breda, The Netherlands). Starting from total RNA samples, small RNAs were size selected and ligated for sequencing following the TruSeq Small Prep Kit Preparation (15004197 Rev. D, Illumina, Eindhoven, The Netherlands). Samples were sequenced on the HiSeq 2000 (Illumina) in single-end 50 bp. Sequencing data have been deposited on ENA (European Nucleotide Archives) under the accession number PRJEB53387. Small RNA reads were first trimmed from the 5′ adapter sequence, and only the post-trimmed reads of 16 to 35 bp were selected. All trimmed reads were then mapped using Patman [37] against the human precursor database from miRbase (release 11), allowing no mismatches or gaps. Patman outputs were parsed to obtain all read mapping to each miRNA arm (5′ and 3′). Normalization and expression analysis were performed using the R package DESeq2 [38].

### 4.4. Bioinformatics Analysis and Modeling

The analysis initially included miRNAs commonly detected in both liver and serum samples. A data clean-up step was carried out to improve the robustness of the analysis. Accordingly, miRNAs detected in <90% or <82.5% of the liver and serum samples, respectively, as well as those miRNAs showing median reads higher in serum than in liver, were removed, leaving a set of 154 miRNAs for further analysis.

Two independent multivariate partial least squares (PLS) regression methods were built to predict: (a) the concentration of TG in human liver based on liver miRNAs and (b) the steatosis level (% of fat in biopsy images) in human liver based on serum miRNAs. PLS was selected for multivariate regression because of the expected multicollinearity among the covariates (miRNAs) and the large number of miRNAs (i.e., 154) compared to the smaller sample size (i.e., 20–23). The selection of the number of PLS latent variables was based on results obtained by leave-one-out cross validation (LOOCV). The assessment of the significance of the figures of merit (e.g., root mean square error of cross validation, RMSECV), was carried out by permutation testing (500 permutations), where the *p*-value was estimated as the fraction of the permuted models, providing a better (i.e., lower RMSECV) estimate than that estimated using the original y vector.

The recursive feature elimination (RFE) algorithm was employed to find the miRNA most relevant in predicting the target variable (TG concentration or % of fat assessed in the biopsy images). Here, a PLS full model was created, and a measure of variable importance (VIP score) was computed to rank the predictors from most important to least. At each stage of the search, the least important predictor was eliminated prior to rebuilding the model. At each iteration, the PLS model performance was assessed using LOOCV, and a new RMSECV was reported. The analysis of the evolution of the obtained RMSECV during the RFE searches was used for the identification of two subsets of miRNAs for each model providing the optimal model performance. The analyses of the two datasets by PLS-RFE enabled the identification of 50 miRNAs in each of the datasets associated with the liver TG concentration and biopsy fat%, and 19 among them were commonly selected in both cases.

Finally, PLS regression models to predict steatosis grade with 2 or 3 miRNAs were built. The low number of miRNAs included in this step allowed us to evaluate all possible combinations of the list of 19 miRNAs, taken 2 or 3 at a time (i.e., 171 and 969, respectively). The evaluation of these models was based on the RMSECV and on the biological relevance of selected miRNAs in lipid metabolism and NAFLD-associated pathways assessed through an in silico search of mRNA targets.

## 5. Conclusions

In this proof-of-concept study, we demonstrated that it is possible to identify serum miRNAs, with biological significance, that correlate with the % of liver fat in NAFLD patients. Moreover, our findings allowed us to suggest that a panel of biomarker miRNAs, identified according to the strategy of this study, could become a promising tool to detect and stratify patients with NAFLD through a minimally invasive procedure. Thus, algorithms based on circulating miRNAs could facilitate a “next level” quantitative diagnosis and clinical follow-up of NAFLD patients, even at early stages of the disease.

## Figures and Tables

**Table 1:** Pearson correlation coefficients (R) and biological significance of 19 selected miRNAs.

miRNA	Guide Passenger (1/0)	Liver R (*p*-Value)	Serum R (*p*-Value)	Lipid Metab. Target Genes		L/S Ratio *	Penalty Score
				mirPath microTcds	mirDIP Target Scan		
miR-10a-5p	1	0.61 (0.003)	−0.65 (0.0007)	18	20	99	0
miR-98-5p	1	−0.49 (0.02)	0.74 (0.0001)	29	39	48	0
miR-19a-3p	1	−0.56 (0.008)	0.61 (0.001)	36	39	63	0
miR-30e-5p	1	−0.61 (0.003)	0.44 (0.03)	40	31	12	0
miR-32-5p	1	−0.45 (0.03)	0.51 (0.01)	42	36	14	0
miR-145-5p	1	0.62 (0.002)	−0.54 (0.007)	8	23	104	−1
let-7d-5p	1	0.64 (0.002)	0.49 (0.02)	15	43	2	−1
miR-181c-5p	1	0.70 (0.0003)	−0.47 (0.02)	12	41	6	−1
miR-23a-3p	1	0.70 (0.0004)	−0.47 (0.02)	36	34	6	−1
let-7b-5p	1	0.61 (0.003)	0.43 (0.04)	29	41	4	−1
miR-148a-3p	1	−0.59 (0.004)	0.23 (0.3)	31	51	331	−1
miR-191-5p	1	0.47 (0.03)	−0.49 (0.01)	0	2	3	−2
miR-769-5p	1	0.69 (0.0005)	−0.47 (0.02)	3	0	6	−2
mir-140-3p	1	0.68 (0.0006)	−0.41 (0.05)	19	0	5	−2
mir-660-5p	1	−0.58 (0.005)	−0.40 (0.06)	8	0	21	−2
miR-335-5p	1	−0.29 (0.2)	0.28 (0.2)	9	22	11	−3
mir-30a-3p	0	0.56 (0.007)	0.58 (0.003)	8	0	189	−3
miR-136-3p	0	−0.62 (0.002)	0.42 (0.04)	3	0	62	−3
miR-17-3p	0	−0.51 (0.02)	−0.17 (0.4)	17	0	11	−5

Note: *: Ratio of mean values in liver and serum samples.

**Table 2:** Best serum miRNA combinations predicting fat % with an error <5% of fat. Values in parentheses correspond to the coefficients (autoscaled intensities, C1, C2, C3) in the corresponding PLS regression vector formula (fat% = miRNA1 × C1 + miRNA2 × C2 + miRNA3 × C3).

RMSECV	miRNA 1	miRNA 2	miRNA 3
4.4	miR-98-5p (2.55)	miR-19a-3p (2.25)	miR-145-5p (−2.00)
4.4	miR-98-5p (2.81)	miR-32-5p (2.05)	miR-145-5p (−2.20)
4.5	miR-30e-5p (1.73)	miR-98-5p (3.14)	miR-145-5p (−2.46)
4.5	miR-98-5p (3.75)	miR-145-5p (-2.93)	
4.6	miR-98-5p (3.75)	miR-32-5p (2.73)	
4.7	miR-98-5p (3.13)	miR-19a-3p (2.76)	

**Table 3:** Baseline characteristics of the donor study cohort (liver).

	Mean ± SD	Min	Max	CV%
µg Liver TG/mg prot	919 ± 494	210	1948	60%
µg Liver lipids/mg prot	730 ± 539	107	1894	70%
Age	57 ± 14	21	75	26%
Weight (Kg)	82 ± 13	60	110	16%
Height (cm)	171 ± 6	157	185	4%
Body mass index (kg/m^2^)	28 ± 5	21	38	16%
Thorax (cm)	107 ± 13	84	134	13%
Abdomen (cm)	107 ± 12	86	127	12%
Bilirubin (mg/dL)	0.8 ± 0.7	0.1	2.8	81%
Creatinine (mg/dL)	1.0 ± 0.3	0.4	1.6	32%
Glucose (mg/dL)	185 ± 70	84	340	39%
AST (U/L)	38 ± 20	18	103	55%
ALP (U/L)	32 ± 22	13	94	72%
Hemoglobin (g/dL)	11 ± 3	4	15	26%
Urea (mg/dL)	47 ± 17	25	77	36%
K + (mEq/L)	3.9 ± 0.5	3.3	5.1	12%
Na + (mEq/L)	150 ± 13	136	192	9%
QUICK index	81 ± 17	40	100	22%

**Table 4:** Baseline characteristics of the NAFLD study cohort (serum).

		Mean ± SD or nº of Cases (%)
Age (years)		51 ± 11
Sex: Male—Female		11 (48%)–12 (52%)
Body mass index (kg/m^2^)		31 ± 6
Glucose (mg/dL)		116 ± 40
TG (mg/dL)		154 ± 67
Total cholesterol (mg/dL)		185 ± 27
HDL-cholesterol (mg/dL)		45 ± 16
LDL-cholesterol (mg/dL)		106 ± 29
Total bilirubin (mg/dL)		0.6 ± 0.3
Albumin (g/dL)		4.6 ± 0.2
Platelets (10^3^/µL)		276 ± 90
ALT (IU/L)		50 ± 33
AST (IU/L)		43 ± 33
ɣ-GT (IU/L)		84 ± 61
ALP (IU/L)		83 ± 35
Prothrombin (s)		14 ± 2
Hemoglobin (g/dL)		14 ± 1
Transferrin saturation (%)		27 ± 14
Insulin (µU/mL)		22 ± 15
Steatosis (%)		
	Grade 0	6 (26%)
	Grade 1	6 (26%)
	Grade 2	6 (26%)
	Grade 3	5 (22%)
Ballooning (%)	None (0)	11 (48%)
	Moderate (1)	9 (39%)
	Severe (2)	3 (13%)
Lobular inflammation (%)	None (0)	8 (35%)
	Moderate (1)	13 (56%)
	Severe (2)	2 (9%)
Fibrosis (%)	Stage 0	12 (53%)
	Stage 1	7 (30%)
	Stage 2	1 (4%)
	Stage 3	3 (13%)
	Stage 4	0 (0%)
NAS scores (%)	NAS: 0–2	12 (53%)
	NAS: 3–4	4 (17%)
	NAS: 5–8	7 (30%)
SAF activity scores (%)	A: 0–1	13 (57%)
	A: 2–3–4	10 (43%)

## Data Availability

The miRNA sequencing datasets supporting the conclusions of this article have been deposited on ENA (European Nucleotide Archives) under the accession number PRJEB53387.
